# Randomized controlled trial of multidisciplinary team stress and performance in immersive simulation for management of infant in shock: study protocol

**DOI:** 10.1186/s13049-016-0229-0

**Published:** 2016-03-25

**Authors:** Daniel Aiham Ghazali, Stéphanie Ragot, Cyril Breque, Youcef Guechi, Amélie Boureau-Voultoury, Franck Petitpas, Denis Oriot

**Affiliations:** Emergency Department and Emergency Medical Service, University Hospital of Poitiers, 2 rue de la Miletrie, Poitiers, 86000 France; INSERM—CIC1402, University Hospital of Poitiers, 2 rue de la Miletrie, Poitiers, 86000 France; Simulation Laboratory, Faculty of Medicine, University of Poitiers, 6 rue de la Miletrie, Poitiers, 86000 France; Pediatric Emergency Department, University Hospital of Poitiers, 2 rue de la Miletrie, Poitiers, 86000 France; Surgical Critical Care Unit, University Hospital of Poitiers, 2 rue de la Miletrie, Poitiers, 86000 France

**Keywords:** Randomized controlled trial, Simulation, Multidisciplinary team, Performance, Stress

## Abstract

**Background:**

Human error and system failures continue to play a substantial role in adverse outcomes in healthcare. Simulation improves management of patients in critical condition, especially if it is undertaken by a multidisciplinary team. It covers technical skills (technical and therapeutic procedures) and non-technical skills, known as Crisis Resource Management. The relationship between stress and performance is theoretically described by the Yerkes-Dodson law as an inverted U-shaped curve. Performance is very low for a low level of stress and increases with an increased level of stress, up to a point, after which performance decreases and becomes severely impaired. The objectives of this randomized trial are to study the effect of stress on performance and the effect of repeated simulation sessions on performance and stress.

**Methods:**

This study is a single-center, investigator-initiated randomized controlled trial including 48 participants distributed in 12 multidisciplinary teams. Each team is made up of 4 persons: an emergency physician, a resident, a nurse, and an ambulance driver who usually constitute a French Emergency Medical Service team. Six multidisciplinary teams are planning to undergo 9 simulation sessions over 1 year (experimental group), and 6 multidisciplinary teams are planning to undergo 3 simulation sessions over 1 year (control group). Evidence of the existence of stress will be assessed according to 3 criteria: biological, electrophysiological, and psychological stress. The impact of stress on overall team performance, technical procedure and teamwork will be evaluated. Participant self-assessment of the perceived impact of simulations on clinical practice will be collected. Detection of post-traumatic stress disorder will be performed by self-assessment questionnaire on the 7^th^ day and after 1 month.

**Discussion:**

We will concomitantly evaluate technical and non-technical performance, and the impact of stress on both. This is the first randomized trial studying repetition of simulation sessions and its impact on both clinical performance and stress, which is explored by objective and subjective assessments. We expect that stress decreases team performance and that repeated simulation will increase it. We expect no variation of stress parameters regardless of the level of performance.

**Trial registration:**

ClinicalTrials.gov registration number NCT02424890

## Background

### Performance, stress, and coping mechanisms

Human error and system failures substantially contribute to adverse outcomes in health care [1]. The safety of a patient in vital distress depends on coordinated actions of providers in an algorithm defined by international recommendations [2, 3]. Performance, i.e. overall quality of care, requires that team leader and members have sufficient knowledge and master procedural skills [4], and that they effectively communicate [5] by developing non-technical skills [6]. Improved team performance and reduction of errors during teamwork have been reported in Emergency Medicine for several decades [7]. Simulation improves management of patients in critical condition, especially if it is undertaken by a multidisciplinary team (MDT) [1, 8] in adult [9] or pediatric [10, 11] cases. Systematic team training improves performance [12] and patients’ safety [13] and correlation between non-technical skills and clinical performance has been established [14]. Non-technical skills are known as CRM—Crisis Resource Management –, which includes task management, teamwork, situational awareness, and decision-making [15]. Some of the CRM assessment tools used in simulation were reported by Selvadilis [16]. Simulation-based training should focus on leadership as a target because it could improve many team processes and performance [17]. Emergency teams face unexpected and unpredictable situations requiring prompt decision-making, and may develop poor management of life-threatening events because of stress [18].

Excessive stress impairs performance [19]. Stress is ‘the non-specific response of the body to any demand for change’ [20], defined as a ‘state of real or perceived threat to homeostasis’ [21]. Stressors, as threats, activate the endocrine, nervous, and immune systems, known as stress response [22]. So, stress can be measured both subjectively and objectively. It refers to psychological (perceived stress), biological, and electrophysiological modulation due to an aggression of the organism causing an emotional response—particularly acute anxiety—and impairment of cognitive processes [23, 24]. The relationship between stress and performance is described as an inverted U-shaped curve [25]. Performance is very low for a low level of stress and increases with an increased level of stress, up to a point, after which performance decreases and becomes severely impaired [26]. The Yerkes-Dodson law is applicable to technical skills in simulation. Positive association between stress and performance in high-fidelity simulation-based training has been decribed [27], whereas excessive stress impairs technical performance [28–30]. Stress also impairs all CRM principles [31] as well as attention, memory, decision-making, and group performance [18]. It can lead to human errors and decrease recognition of these errors [32]. Excessive stress impairs team capabilities like communication, as the leader becomes less receptive to suggestions and fails to share the mental model [33]. When stress is intense or repeated, it might expose providers to a psychological impact [34] and the risk of post-traumatic stress disorder (PTSD) [35, 36]. PTSD usually occurs between 1 week and 1 month after a psychologically traumatic event [37], characterized by pathognomonic repetition syndrome and other non-specific symptoms.

Acute stress leads to coping mechanisms [38]. It has been shown that poor management of stress has a negative impact, particularly on performance [39]. In simulation, surgeons’ stress and coping are likely to influence surgical performance [29]. However, even if the relationship between stress, coping and performance has been widely studied, to our knowledge there is no study describing concomitant changes in performance and stress during repetitive simulations. Do repeated simulations increase performance and reduce stress, or is there increased performance with the same level of stress, which would suggest coping and shift the Yerkes-Dodson curve to the left? Contradictory findings have been published on subjective and objective parameters of stress: correlation between the State-Trait Anxiety Inventory (STAI) scores and salivary amylase levels [40] but not with salivary cortisol (SC) levels [41], differences between perceived stress and objective stress measured by heart rate (HR), respiratory sinus arrhythmia, and SC [42]. Consequently, using a combination of perceived and physiological markers of stress may be more reliable than using a single measurement. Concomitant changes in objective and subjective stress parameters have been poorly studied during repeated simulation sessions. Furthermore, there is no data on the possible occurrence of a PTSD after simulation session(s) and its impact on performance whereas simulated emergency is likely to be more stressful than a similar case in real life [43].

### Rationale for a model of infant shock

Team training should improve management of infant shock as previously suggested [44, 45]. Moreover, life-threatening situations are less frequent in pediatric than in adult emergencies. Likewise, requirements for ethics may be stronger in pediatrics than in other fields of healthcare [46], which leads to high error rates to management of children in exceedingly busy and stressful workplaces [47]. Given this context, a model for infant shock may facilitate assessment of a significantly enhanced performance by repeated simulations in a stressful environment in which stress parameters are recorded during sessions. Inasmuch as it is supposed to generate high stress, this model should optimize analysis of the benefit of repeated simulations and the relationship between stress and performance.

### Hypotheses and aim of the study

We hypothesize that compared to three simulation sessions, nine simulation sessions over 1 year will have a greater effect on MDTs’ technical and non-technical performance and reduce stress. We speculate that high-fidelity simulation can generate a state of acute stress (identified by objective parameters of stress) without generating post-traumatic stress disorder because the scenarios have been designed to be appropriately stressful and are followed by systematic standardized post-scenario debriefing.

To investigate some of the non-elucidated points in immersive simulation researches, the aim of this study is to explore the effect of stress on performance and the effect of repeated simulation sessions on performance and stress, with three objectives: evidence of stress, evolution of performance under stress, and evolution of stress and performance during repeated simulations.

## Methods and design

### Design

**Fig. 1:**
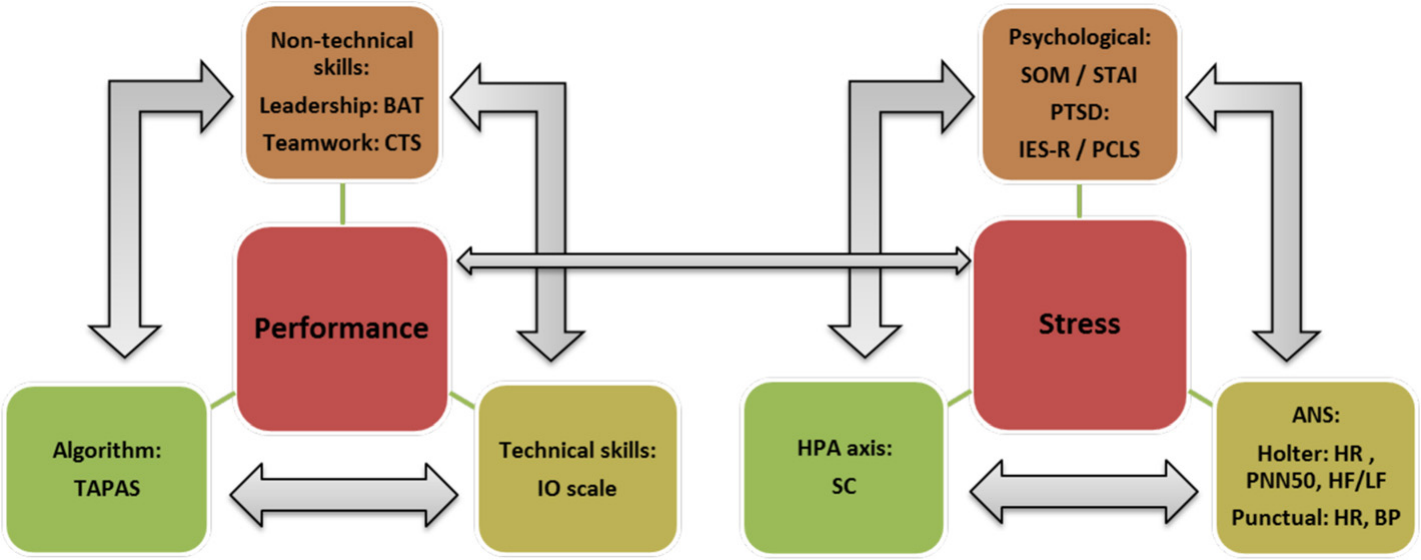
Different components of the intervention and potential correlations. ANS: autonomic nervous system; BAT: Behavioral Assessment Tool; BP: blood pressure; CTS: Clinical Teamwork Scale; HPA: hypothalamic pituitary adrenal stress axis; HF/LF: high frequency / low frequency ratio; HR: heart rate; IES-R: Impact of Event Scale-Revised; IO: intra-osseous; PCLS: Post-Traumatic Check-List Scale; PNN50: proportion of successive NN that differ by more than 50 ms divided by total number of NN; PTSD: post-traumatic stress disorder; SC: salivary cortisol; SOM: Stress-O-Meter; STAI: State Trait Anxiety Inventory; TAPAS: Team Average Performance Assessment Scale.  Potential correlation

The design is a single-center, investigator-initiated randomized controlled trial. The study was scheduled from September 2013 to December 2015, including 12 months for the simulation sessions (December 2014 to December 2015), and followed by a data management period. Performance and stress are assessed and correlation between all the components of performance and stress, and between stress and performance will be analyzed (Fig. 1).

### Setting and participants

The trial is being undertaken in the Laboratory of Simulation SiMI—INSERM 1402, Faculty of Medicine, University of Poitiers, France. Each MDT is made up of 4 persons: an emergency physician, a resident, a nurse, and an ambulance driver who usually constitute Emergency Medical Service team in France. All emergency physicians with less than 7 years of experience working in an Emergency Department of one of the hospitals in the Poitou-Charentes region (1.8 10^6^ inhabitants) were eligible for inclusion in the trial. All healthcare providers (nurses and ambulance drivers) from the Emergency Medical System of the University Hospital of Poitiers were likewise eligible.

Inclusion criteria are: participation on a voluntary basis; being more than 18 years old; informed consent for research and video.

The constitution of a team of 4 persons relies on: 1) Emergency physicians from the Poitou-Charentes area, having obtained the University Diploma of Pediatric Emergency Procedures (University of Poitiers, France) after issue of the 2010 guidelines by the American Heart Association [2] and the European Resuscitation Council [3]; 2) Residents specialized in Emergency Medicine, trained in pediatric emergency procedures: clinical training in a Pediatric Emergency Unit and/or the university course; 3) Nurses belonging to the Emergency Medical Service of the University Hospital of Poitiers, with less than 7 years of experience and having obtained the European Pediatric Immediate Life Support degree over the past 2 years; 4) Ambulance drivers belonging to the Emergency Medical Service of the University Hospital of Poitiers, with less than 7 years of experience.

Non-inclusion criteria are: pregnant women; past history (any disease that could induce modifications related to stress, or worsen in relation to stress) and/or psychiatric disease modifying stress response; cardiac or neurological history with convulsions; pacemaker or implantable defibrillator; treatment with medication having a potential effect on stress parameters: cardiotropic agents, β2-agonist bronchodilators, steroids, hormone replacement therapy, and psychotropic agents.

This study was considered as a biomedical research on healthy volunteers by the Agence Nationale de Sécurité du Médicament (National Medication Safety Agency) and registered under the number 2013-A00648-37. The research site (Simulation Laboratory of the Faculty of Medicine of Poitiers, France) was approved by the Agence Régionale de la Santé (Health Regional Agency) of the Poitou-Charentes region of France. The study protocol, information form, and consent form were approved by the Comité de Protection des Personnes III de la region Ouest (Western France Person Protection Committee III) and were registered under the number 13.05.16. Furthermore, the registration number from ClinicalTrials.gov (a WHO-approved primary registry) is NCT02424890 [48].

### Recruitment

**Fig. 2:**
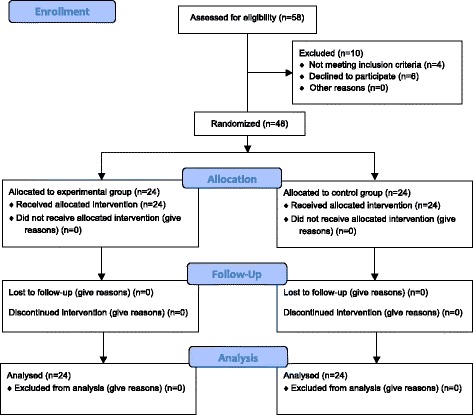
CONSORT 2010 Flow Diagram

Strict inclusion criteria were used to obtain a homogeneous professional experience and training of participants, whatever their status. For each status, an exhaustive list of personnel was used for sampling. Because of an estimated refusal rate of 50 %, we considered 24 persons of each status to be interested in the study. Participants were randomly chosen and contacted by email for presentation of the study and consent to participate. In case of agreement, a final consent form was signed before the first session. Twelve participants for each status were drawn by lots by the trial coordinator among each status population (until all consented) and randomized to form different teams. Twelve MDTs of 4 persons were constituted. Participant recruitment, treatment and analysis throughout the trial are reported on Fig. 2 [49]. A second randomization was performed on the 12 MDTs by the methodologist to obtain the two groups: an experimental group constituted by 6 MDTs to undertake a simulation session every 6 weeks, i.e., 9 simulation sessions over 1 year, and a control group constituted by 6 MDTs to undertake a simulation session every 6 months, i.e., 3 simulation sessions over 1 year. This latter group constituted the active comparator.

### Intervention

**Fig. 3:**
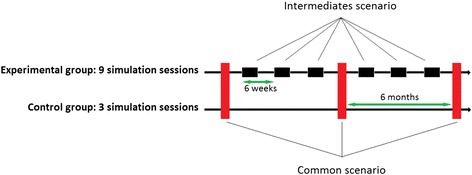
Repetition of simulation sessions over one year

**Fig. 4:**
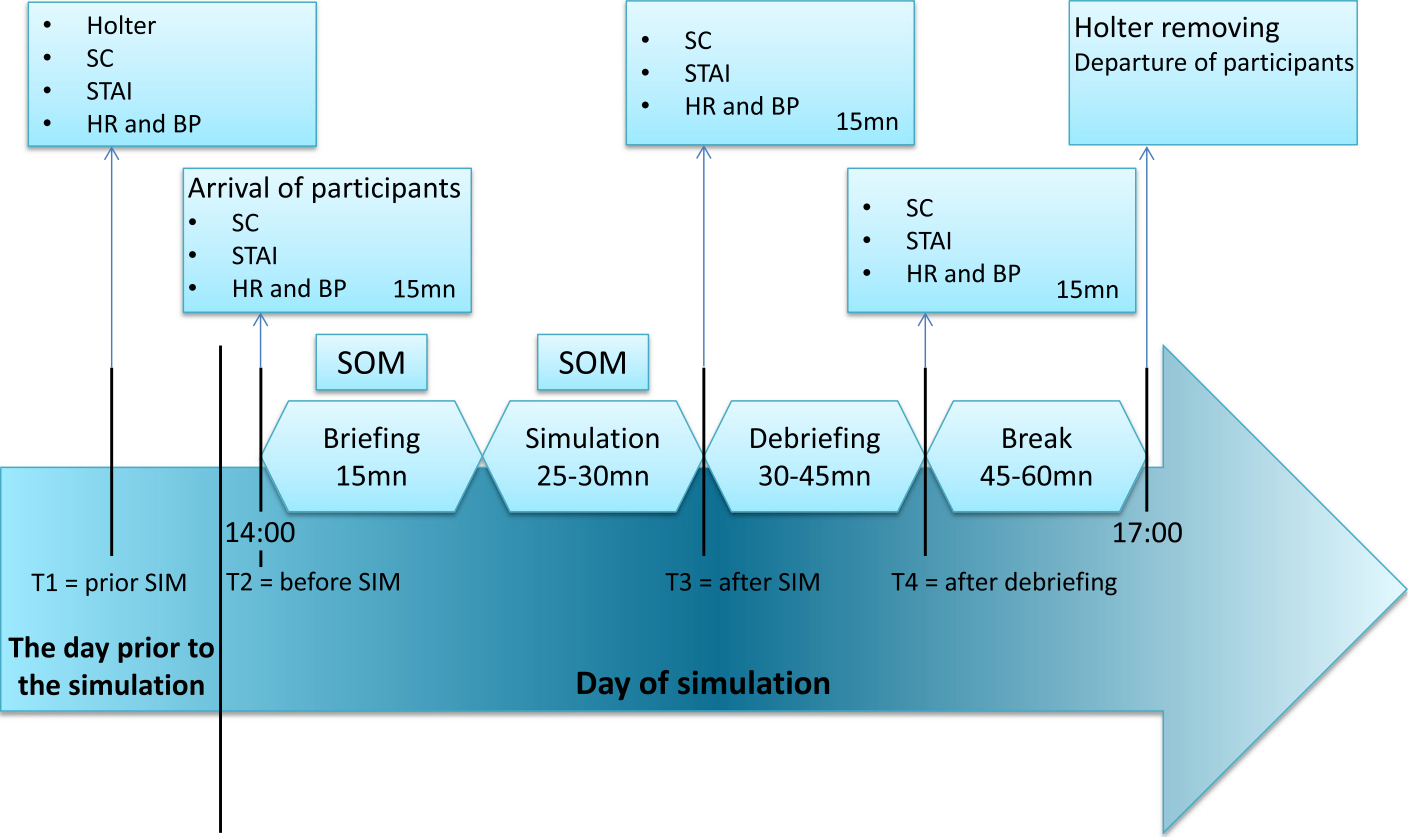
Course of a simulation session. BP: blood pressure; HR: heart rate; SC: salivary cortisol; SOM: Stress-O-Meter; STAI: State Trait Anxiety Inventory

The repetition rate of simulation sessions is the only varying component between the 2 randomized groups (9 or 3 simulation sessions over 1 year) (Fig. 3). The scenarios were designed using a typology of simulation, which incorporates three elements of fidelity in simulation: environmental, equipment, and psychological fidelity [50]. A high-fidelity manikin (SimNewB*, Laerdal®) from the Laboratory of Simulation of Poitiers is used to illustrate scenarios of infant shock with requirement of IO access insertion. Nine scenarios were drawn by lots among 18 cases of infant shock: 4 hypovolemic shocks, hemorrhagic shock in severe trauma, anaphylactic shock, 2 cardiogenic shocks, and septic shock. Prior to the research protocol, all emergency physicians had identical training in insertion of intra-osseous (IO) access in infants with performance assessment on the validated scale for simulated IO insertion developed in our Simulation Laboratory [51]. Because there exists no scale to assess clinical performance of emergency teams, our Simulation Laboratory designed and validated a team average performance assessment scale (TAPAS). Psychometric characteristics of TAPAS were calculated (publication submitted). Non-technical skills are assessed by The Clinical Teamwork Scale (CTS) for teamwork and CRM [52], and by the Behavioral Assessment Tool (BAT) for leadership [53]. All sessions are scheduled the same day of the week at 2:00 pm because of the circadian cycle of cortisol. Each simulation—lasting 25–30 min—is preceded by a briefing (15 min), and followed by a “good judgment” debriefing (30–45 min) [54]. The purpose of debriefing is to improve professional performance through facilitated (supervisor) recognition and closure of gaps in performance [55]. Moreover, three periods of 15mn are dedicated to saliva samples and data collection (HR, BP, and STAI). Then there is a 45–60mn “snack break” lasting until 5:00 pm including participants, supervisors, and the investigator to allow physiological variables to return to normal conditions (Fig. 4). During the simulation, stressful conditions are related to different sources: scenarios themselves (hypoxia, worsening of shock, seizures, cardiac arrest), realistic environment (beeps and alarms), and the untimely irruption of simulated parents in the Emergency Room according to each scenario. Stress is assessed in 3 ways: psychological, biological and electrophysiological (Fig. 4). We considered stress assessment methods that were compatible with simulated team management of life-threatening events. Self-reporting of stress applies various scales: the Stress-O-Meter (SOM) [56] and the State-Trait Anxiety Inventory (STAI) [57], commonly used in simulation [29, 58]. PSTD is detected by the Impact of Event Scale-Revised (IES-R) on the 7^th^ day after the event [59, 60] and the Post-traumatic Check-List Scale (PCLS) 1 month later [61]. Electrophysiological stress is assessed by HR and heart rate variability (HRV) in time and frequency domain from Holter data, and timely measurements of HR and BP. Temporal and spectral analysis of HRV [62] is based on collection of a continuous signal beat-to-beat RR interval (or NN interval, i.e. normal to normal) detected on electrocardiography (ECG) and its decomposition through fast Fourier transform. Time-domain method was based on the the number of interval differences of successive NN intervals greater than 50 ms and the analysis of PNN50 (the proportion of successive NN that differ by more than 50 ms divided by total number of NN) [63]. Spectral analysis (frequency domain) can differentiate the two components of cardiac autonomic nervous system: parasympathetic nerve activity, by measurement of “high frequency” (HF) spectral powers, and sympathetic nerve activity by the “low frequency” (LF)/HF ratio, also known as “sympathovagal balance” [64]. Biological stress is assessed by a non-invasive measurement of SC, a well-established biomarker of stress used in simulation [19, 65, 66].

All sessions are videotaped in order to replay them for assessment if necessary. Two independent observers (among a group of 8) evaluate each simulation session. They work in the Simulation Laboratory of the Faculty of Medicine of Poitiers and are randomly chosen. All were trained in simulation and debriefing. They assess overall team performance with respect for the algorithm and therapy, insertion of the IO access, and CRM. Mean scores between the two observers will be used as the reference value.

### Outcome measures

The allocation was concealed from the two independent observers assessing participants and data managers. Participants are not blinded to the intervention. Data recording will be carried out after the end of all simulation sessions to avoid bias related to knowledge of data by the investigator. Table 1 provides the different evaluation times and data collection on stress. Table 2 provides an overview of variables and outcomes. It is inspired by SPIRIT 2013 guidance for clinical trial protocols [67]. The analysis will focus on: 1 primary outcome and 2 secondary outcome measures.

**Table 1:** Time schedule of measurements

Variables		Day prior	Before Sim	Sim	Post Sim	Debrief	Post debrief	H + 2	1 week	1 month
Performance	Global performance			X						
	IO access			X						
	Leadership (BAT)			X						
	Teamwork (CTS)			X						
Stress parameters	Salivary cortisol	X	X		X		X			
	Holter parameters	X	X	X	X	X	X	X		
	BP HR	X	X		X		X			
	SOM		X	X						
	STAI scale	X	X		X		X			
	EIS-R scale								X	
	PCLS scale									X

Legend: *BAT* Behavioral Assessment Tool, *BP* blood pressure, *CTS* Clinical Teamwork Scale, *EIS*-*R* Impact of Event Scale-Revised, *HR* heart rate, *IO* intra-osseous, *PCLS* Post-traumatic Check-List Scale, *SOM* Stress-O-Meter, *STAI* State Trait Anxiety Inventory

**Table 2:** Variables, research hypothesis, outcome measures and methods of statistical analysis

Measures	Research hypothesis	Variables and outcome measures		Population	Statistical test
Descriptive analysis	Homogeneity of groups	Quantitative variables	Scores (/100), SC (μg/dl), HR (/mn), BP (mmHg), PNN50 (%), HF/LF	Total population	mean, standard deviation and / or median and interquartile range
		Qualitative variables	age, sex, status, years of experience	Total population	Number and percentage
Evaluation of the effect of stress on performance	Impact of stress on performance with capabilities(stress adapted) or decreased (unsuitable stress)	Markers of biological stress (SC)		Total population	Pearson correlation coefficient (or Spearman correlation coefficient, if necessary)
		Markers of electrophysiological stress (HR, BP, PNN50, HF/LF)		Groups 1 and 2	
		Markers of psychological stress (STAI, PCLS, IES-R)		Research of heterogeneity related to status	
		Performance: overall performance scores, IO access score and scales assessing teamwork			
Evaluation of changes in performance scores over time	Performance scores improved over time	Overall team performance, IO access performance score and scales assessing teamwork		Groups 1 et 2	ANOVA for repeated measures (or a non-parametric test like Kruskal-Wallis if necessary). Scheffe tests to test the difference by pair in case of significance with the ANOVA test
		For the whole population, linear models with mixed effects may be considered in order to take into account in the same analysis all data collected in the simulation sessions, including the development of stress management strategies in parallel to stress and repeated simulations.			
Evaluation of repeated simulations on performance	Improvement of performance during repeated simulations with higher scores in group 1	Score of overall performance, IO access score and score of scales assessing teamwork:		Comparison between group 1 and 2	Student *t*-test and research of status and group effect by ANVOA
		Relative variation of the different scores = (final score—T0 score)/T0 score			
Evaluation of repeated simulations on stress level	Repetitive simulation-based training-related improvement in performance is not associated with a blunting of stress response in MDT members	Markers of biological stress (SC)		Comparison between group 1 and group 2	Student *t*-test or non-parametric test U of Mann-Whitney if necessary
		Markers of electrophysiological stress (HR, BP, PNN50, HF/LF)			
		Markers of psychological stress (STAI, PCLS, IES-R)			
Inter-observer reproducibility	Very good reproducibility due to the use of validated scales	Scales of assessment:		Observers	Intra-class coefficient correlation
		Overall performance, IO access, BAT, CTS			

Legend: *BAT* Behavioral Assessment Tool, *BP* blood pressure, *CTS* Clinical Teamwork Scale, *EIS*-*R* Impact of Event Scale-Revised, *HF* high frequency, *HR* heart rate, *IO* intra-osseous, *LF* low frequency, *PCLS* Post-traumatic Check-List Scale, *PNN50* proportion of successive NN that differ by more than 50 ms divided by total number of NN, *SOM* Stress-O-Meter, *SC* salivary cortisol, *STAI* State Trait Anxiety Inventory

#### Primary outcome measure: Evidence of the existence of stress

Acute stress immediately acts on the autonomic nervous system [68], resulting in a massive release of norepinephrine in sympathetic nerve endings, and leading to tachycardia and increased blood pressure (BP). The most prolonged somatic responses to stress involve the adrenal cortical axis [69], releasing ACTH and increasing cortisol. There exist many types of stress assessment in simulation studies [70], which often use a combination of physiological markers [33], such as increased heart rate (HR) [65, 71], and BP [72], modification of HRV, increased skin conductance level [73], and increased number of eye blinks (electrooculogram) [74]. Hormones modified by stress can be measured in saliva: SC [75, 76], and salivary alpha amylase [40, 77, 78]. All participants undergo significant stress during immersive simulation [65] and perceived-stress is commonly assessed in simulation [70] based on a questionnaire [57, 79, 80] or on a self-report score [81]. However, to our knowledge, the occurrence of PTSD has not been searched during repetitive simulations. Throughout the scenarios of this study, evidence of stress is assessed in 3 ways: biological stress (SC), electrophysiological stress (Holter 24 h and punctual measures), and psychological stress. SC is measured by an ELISA kit (IBL international®, Hamburg, Germany). The microtitter plate is read at 450 nm. Holter parameters (HR, PNN50 and the ratio LF/HF) are obtained with the software Synscope® (Sorin Group®) during 24 h recording, starting the day prior to the simulation until the break after simulation. Timely measures of HR and BP are associated with this analysis. Psychological stress is assessed by self-evaluation (SOM self-rating scale, STAI) after the simulation in a calm room where participants are seated. Many scales exist to detect PTSD [59–61, 68, 82–84]. In the present study IES-R is e-mailed to participants on day 7^th^ [60] and PCLS at 1 month [61] to detect occurrence of PTSD.

#### Secondary outcome measures

##### Evaluation of the effect of stress on performance

Impact of stress on performance is assessed in three ways: TAPAS score (submitted for publication) for overall technical performance, IO access performance assessment score [51], and non-technical performance by BAT score for leadership assessment [53] and CTS score for CRM assessment [52].

##### Evaluation of the effect of repetition of simulation sessions

The evaluation of the effect of repeated stimulation sessions will be carried out by comparison of experimental group (9 simulation sessions over 1 year) versus control group (3 sessions over 1 year) (Table 2). It will be evaluated for team performance using the same assessment tools: TAPAS, IO access performance assessment scale, BAT, and CTS. The effect of repeated simulation sessions on stress will be investigated through variation of the same stress markers and by studying the development of coping. This will allow us to determine whether repeated simulation sessions, which are expected to improve performance, are actually occurring with or without a high level of stress. Occurrence of coping strategies will be investigated by the evolution of BAT and CTS scores despite high level of stress. Participant self-assessment will be requested at 6 and 12 months after the end of simulations exploring levels 1, 2, and 3 of the Kirkpatrick model [85].

### Statistical analysis

The number of required subjects was calculated to meet the primary objective of the study: evidence of a relationship between stress and performance. We consider a relationship between stress and performance to be significant if the correlation coefficient R^2^ reaches a minimum value of 0.50. For a risk of the first kind at 5 %, with a power of 90 % and a bilateral situation, the number of subjects to be included was calculated at 48 (Proc POWER, SAS). We included 12 MDTs, each of them including 1 emergency physician, 1 resident, 1 nurse, and 1 ambulance driver. This will enable us to find a difference of 2.1 points in the IO access performance assessment score (standard deviation of 1.02) [51]. A *p* <0.05 will be considered statistically significant.

All data will be transformed to a 100 basis, kept anonymous, and analyzed with Statview version 4.5 (SAS Institute Inc., Cary, NC). Intra-class correlation coefficient will be calculated for assessment scales. Statistical analyses are given on Table 2. Quantitative variables will be described as mean, standard deviation and/or median and interquartile range. Categorical variables will be summarized by raw numbers and percentage. Relationship between stress parameters themselves and with performance scores will be assessed on the whole population and within each group with Pearson (or Spearman) correlation coefficient. Variations of performance scores will be evaluated over time by ANOVA for repeated measures (or Kruskal-Wallis test) in each group. Relative variation will be calculated as different scores ((final score—baseline score)/baseline score) to evaluate repeated simulations on performance. Comparison between groups will use Student *t*-test. We will look for a status effect using ANOVA. A *p* value <0.05 will be considered significant.

## Discussion

### Strengths

The originality of this study resides in three points: 1) Stress is explored by subjective and objective assessments at different times of a high-fidelity simulation session, and stress response after debriefing as well as PTSD had never previously been studied; 2) Concomitant evaluation of technical and non-technical performance, and the impact of stress on both; 3) Finally, this is the first randomized trial studying repeated simulation sessions and their impact on both clinical performance and stress. These assessments are carried out on complete real French Emergency Medical Service teams. Few studies have been performed on real teams [86]. With regard to stress pathways, an exhaustive analyze is done, including hypothalamic pituitary adrenal axis, autonomic nervous system, and self-perceived stress as previously suggested to study stress response [87]. To our knowledge this is the first simulation study addressing the ECG signal by spectral analysis (LF/HF), allowing a more thorough approach to stress. Perceived-stress is punctually studied in simulation immediately after the session [70] with usually high level of stress [65]. However the psychological impact of repeated stressful simulations has not yet studied. We will report the first study of a potential PTSD in medical simulation domain. The results of this study will provide important findings: we expect that stress decreases performances based on technical and non-technical skills and that repeated simulations increase performance. This study will determine in which field repeated simulation sessions are accompanied by an improvement in performance (procedure, teamwork, leadership), and when it occurs.

### Limitations

We are aware of the limitations of this protocol. The number of required subjects was calculated to provide evidence of a relationship between stress and performance. However, some comparisons may be rendered difficult due to large inter-individual variability in terms of both performance and stress. It is consequently difficult to compare groups in terms of leadership (6 vs. 6) whereas it seems easier to compare teams including all participants (24 vs. 24). However, we will be able to correlate whole teams’ performance (technical and non-technical skills) to the leaders’ performance. To our knowledge, no previous study has reported such correlations. It will also be possible to analyze and to correlate changes in absolute and relative variations of stress and performance over time. As regards stress response, paramedics of the French Emergency Medical System have usually spent several years in other departments (anesthesiology or emergency) before recruitment. Therefore, they are older than medical staff with the same level of experience. We have supposed that this age difference will not impact stress parameters [88]. The real challenge is the respect of schedules so as to avoid influencing the stress parameters. We obtained the consent of participants prior to their inclusion in the protocol to respect the comprehensively planned dates of simulations.

### Discussion of study design

After an initial simulation course, skills are preserved in case of retraining [89]; intervals of repetition should range from 6 weeks to 6 months [90]. However, to our knowledge no previous study has defined the optimal frequency of repetition of simulation leading to maximal benefit at minimal cost. Indeed, significant cost and time are known to be associated with high-fidelity simulation training [91]. There is also a lack of homogeneity in the repetition simulation sessions designed to improve procedural skills and team performance [92–94]. With this study design, the two groups cannot be compared throughout the whole period. In fact, comparison can be performed only on a common scenario. As a compromise, an intermediate scenario of the experimental group will be used to analyze the development of skills over time as well as stress parameter evolution. Pre-hospital life-threatening cases of infant with shock are sufficiently rare in clinic for the parameter of repeated simulations to be held solely responsible for observed changes.

### Discussion of primary outcome criterion

A rise in all markers of stress during simulation of MDT management of an infant in shock should be evidenced for all participants [95]. However, literature data suggest a complex mechanism of stress pathways with contradictory conclusions in simulation domain necessitating study of subjective stress and stress response, including the hypothalamic pituitary adrenal axis and the autonomic nervous system. Some authors have found an increase of subjective and objective measures [30], whereas others have found only partial or negligible variation of stress parameters [96]. STAI might increase [66] or decrease [97] in the same field of simulation. Studies on emergency residents have found that self-reported stress increased [98] or did not [99]. There is no data for PTSD assessment in simulation. For objective markers of stress, SC was found to increase [100], to have minor variation [101] or to have no variation [102]. For electrophysiological parameters (HR, HRV, and BP), there is a lack of homogeneity in correlation with other stress parameters: correlation [66], no correlation [103], or partial correlation [41]. Stress response will be studied during simulation but also after debriefing. We have assumed that stress level will increase after simulation and decrease after debriefing using an appropriate technique [69].

### Discussion of the intervention

The model of infant shock was chosen to study stress response and performance in repeated immersive simulation because we expect clear stress response and performance evolution. All scenarios apply the case of a decompensated shock or cardiac arrest to study the same technical skill of IO access, according to the international recommendations [2, 3]. This objectively ensures possible correlation of technical performance between different teams and over time. There is no scenario of respiratory or neurological impairment which reduces the extent of life-threatening emergencies in a child. The field of intervention is consequently focused on only one part of the pediatric emergencies. Nevertheless, diversified clinical situations offer the possibility to have different scenarios with a common objective of IO access. Stress genesis varies in the scenario (monitoring, parental presence, external care-giver…) in order to increase realism. Repeating the same scenario would have been better in terms of focusing on the repetition of scenario as the intervention variable. Nevertheless, it would inevitably have led to a huge bias due to memory retention and creation of automatisms. So the variety of scenarios—none of them were identical—were created to develop MDT management with the same technical skills for IO access and ABCDE approach [104] as well as non-technical skills for CRM and leadership, and to generate stress in different ways.

## Abbreviations

BAT: Behavioral assessment tool
BP: Blood pressure
CRM: Crisis resource management
CTS: Clinical teamwork scale
HF: High frequency
HR: Heart rate
HRV: Heart rate variability
IES-R: Impact of event scale-revised
IO: Intra-osseous
LF: Low frequency
MDT: Multidisciplinary team
PCLS: Post-traumatic check-list scale
PNN50: Proportion of successive NN that differ by more than 50 ms divided by total number of NN
PTSD: Post-traumatic stress disorder
SC: Salivary cortisol
SOM: Stress-o-meter
STAI: State trait anxiety inventory
TAPAS: Team average performance assessment scale
